# Intracellular Aβ pathology and early cognitive impairments in a transgenic rat overexpressing human amyloid precursor protein: a multidimensional study

**DOI:** 10.1186/2051-5960-2-61

**Published:** 2014-06-05

**Authors:** M Florencia Iulita, Simon Allard, Luise Richter, Lisa-Marie Munter, Adriana Ducatenzeiler, Christoph Weise, Sonia Do Carmo, William L Klein, Gerhard Multhaup, A Claudio Cuello

**Affiliations:** Department of Pharmacology and Therapeutics, McGill University, 3655 Sir-William-Osler Promenade, Room 1210, Montreal, Quebec Canada; Department of Anatomy and Cell Biology, McGill University, Montreal, Canada; Department of Neurology and Neurosurgery, McGill University, Montreal, Canada; Intitutes of Chemistry and Biochemistry, Freie Universität, Berlin, Germany; Cognitive Neurology and Alzheimer’s Disease Center, Northwestern University Institute for Neuroscience, Chicago, USA

**Keywords:** Amyloid-β, APP, Intracellular Aβ, Pre-clinical, Alzheimer’s disease

## Abstract

Numerous studies have implicated the abnormal accumulation of intraneuronal amyloid-β (Aβ) as an important contributor to Alzheimer’s disease (AD) pathology, capable of triggering neuroinflammation, tau hyperphosphorylation and cognitive deficits. However, the occurrence and pathological relevance of intracellular Aβ remain a matter of controversial debate. In this study, we have used a multidimensional approach including high-magnification and super-resolution microscopy, cerebro-spinal fluid (CSF) mass spectrometry analysis and ELISA to investigate the Aβ pathology and its associated cognitive impairments, in a novel transgenic rat model overexpressing human APP. Our microscopy studies with quantitative co-localization analysis revealed the presence of intraneuronal Aβ in transgenic rats, with an immunological signal that was clearly distinguished from that of the amyloid precursor protein (APP) and its C-terminal fragments (CTFs). The early intraneuronal pathology was accompanied by a significant elevation of soluble Aβ_42_ peptides that paralleled the presence and progression of early cognitive deficits, several months prior to amyloid plaque deposition. Aβ_38_, Aβ_39_, Aβ_40_ and Aβ_42_ peptides were detected in the rat CSF by MALDI-MS analysis even at the plaque-free stages; suggesting that a combination of intracellular and soluble extracellular Aβ may be responsible for impairing cognition at early time points. Taken together, our results demonstrate that the intraneuronal development of AD-like amyloid pathology includes a mixture of molecular species (Aβ, APP and CTFs) of which a considerable component is Aβ; and that the early presence of these species within neurons has deleterious effects in the CNS, even before the development of full-blown AD-like pathology.

**Electronic supplementary material:**

The online version of this article (doi:10.1186/2051-5960-2-61) contains supplementary material, which is available to authorized users.

## Introduction

Alzheimer’s disease (AD) remains the most common form of age-related dementia, a disorder which affects approximately 36 million sufferers worldwide [1]. Recent studies have revealed the existence of a long asymptomatic phase, where the pathological changes leading to AD begin –at least- decades before the first symptoms of cognitive decline appear [2–4].

Although these studies have addressed the temporal order in which AD pathological hallmarks appear, the exact sequence of cellular and molecular events that lead to AD is still poorly understood. However, it is widely accepted that before the overt deposition of amyloid plaques and neurofibrillary tangles, the accumulation of amyloid-β (Aβ) peptides is one of the first steps in the series of pathogenic changes that lead to neurodegeneration and dementia [5, 6].

The concept of a pre-clinical, asymptomatic phase of AD is gaining increasing support. This is best evidenced by the recent revision of research diagnostic criteria, which now include a new detailed framework for the diagnosis and pre-clinical staging of individuals at-risk [7]. Notably, little is known about this early phase, as most studies of the AD pathology center on moderate-to-late stages. This pre-clinical period deserves further attention, as it should offer a critical window for successful treatment.

Given the lack of definitive AD biomarkers in humans, transgenic animal models of the amyloid pathology continue to be valuable tools to examine molecular changes preceding the deposition of amyloid plaques and associated pathology (i.e. late inflammation, neuritic dystrophy, etc.). For instance, many studies in transgenic models have demonstrated that Aβ accumulates first intraneuronally, before amyloid plaques appear [8–13]; a finding that has been validated in post-mortem AD and Down syndrome (DS) brains [14–20].

A deeper understanding of the pre-plaque stage is of great relevance in view of the fact that the intraneuronal compartment is also a place where Aβ can oligomerize [10, 21, 22] forming toxic aggregates which can impair synaptic plasticity [23–26], induce cognitive impairments [27, 28] and unleash a pro-inflammatory reaction [10, 29]. In fact, the nature and pathological relevance of the intracellularly accumulated material have been questioned in a recent study [30], raising the intriguing possibility that such material is APP, rather than Aβ. The arguments regarding this controversy have been recently reviewed [31]. The idea that the intraneuronal material is only APP is a challenging proposition, and we therefore considered that this issue deserved a thorough, multidisciplinary analysis.

For such study, we have used the McGill-R-Thy1-APP transgenic rat, which is unique compared to other rodent models in that the AD-like phenotype has been achieved with a single genomic insertion of a mutated human APP transgene; minimizing off-target genetic corruption and therefore being closer to the human disease [32]. Our microscopy studies at high magnification and high resolution have revealed a clear segregation between Aβ-immunoreactivity and that of APP and its C-terminal fragments (CTFs). Soluble Aβ_40_ and Aβ_42_ peptides were observed elevated early, in cortex and hippocampus from transgenic rats. The increase in Aβ peptides paralleled the manifestation of cognitive deficits, several months prior to amyloid plaque deposition. Notably, Aβ_38_, Aβ_39_, Aβ_40_ and Aβ_42_ peptides could be detected in the rat cerebro-spinal fluid (CSF) by MALDI-MS analysis, even at plaque-free stages. Taken together, these results indicate that the early, intracellular accumulation of soluble Aβ in McGill transgenic rats is already accompanied by a CNS compromise (i.e. learning and memory impairments). Further to it, we have demonstrated that the early CNS amyloid pathology is reflected and can be monitored in the rat CSF.

## Materials and methods

### Animals

Transgenic rats belonged to the McGill-R-Thy1-APP line, harboring the human APP751 transgene with the Swedish and Indiana mutations under the control of the murine Thy1.2 promoter [11]. The 3-month old group consisted of: transgenic heterozygote (*n* = 7) and homozygote (*n* = 7) rats and non-transgenic (*n* = 10) littermates. The 7-month old group consisted of: transgenic homozygote (*n* = 4) and non-transgenic (*n* = 4) littermates. The 13-month old group consisted of: transgenic heterozygote (*n* = 10) and homozygote (*n* = 6) rats and non-transgenic (*n* = 10) Wistar rats (Charles River, Wilmington, MA), housed in identical conditions. Each group had equal gender representation. Animals were maintained on a 12-hour light/12-hour dark cycle and had *ad libitum* access to water and a standard rodent diet. All procedures were approved beforehand by the Animal Care Committee of McGill University, following the guidelines of the Canadian Council on Animal Care.

### Tissue and CSF collection

Rats were deeply anesthetized with equithesin, and CSF (~80-150 μl) was collected from the cisterna magna using a glass capillary and subsequently frozen at -80°C, following an established protocol [33]. Only clear, blood-free CSF was used for analysis.

For tissue collection, the animals were perfused transcardially with cold saline for 1 min. The brain was removed, one hemisphere was kept for immunohistochemistry (IHC) and the other was dissected, flash-frozen and kept at -80°C for further neurochemical analysis. The hemisphere for IHC was immersion-fixed in cold 4% paraformaldehyde in 0.1 M phosphate buffer (PB) for 24 h and transferred to 30% sucrose in 0.1 M PB. After the brains equilibrated in sucrose, they were cut into 40 μm coronal sections with a freezing microtome (Leica SM 2000R, Germany). Brain sections were stored in cryoprotectant solution (37.5% v/v ethylene glycol, 37.5% w/w sucrose, in PBS pH 7.4) at -20°C until processed for IHC.

### Antibodies

A detailed description of the primary antibodies used in this study can be found in Table 1.

**Table 1:** **List of antibodies used in this study**

Antibody name	Antibody target	Source	Epitope recognized	Obtained from
**McSA1**	Human Aβ	Mouse monoclonal	N-terminus of human Aβ (residues 1–12)	Medimabs, Canada
**MOAB-2**	Human Aβ	Mouse monoclonal	N-terminus of human Aβ (residues 1–4)	Biosensis, Australia
**Nu1**	Human Aβ oligomers	Mouse monoclonal	Conformational, Aβ oligomers	Dr. William Klein, Northwestern University
**pab27576**	APP	Rabbit polyclonal	Last 43 C-terminal residues of APP	Dr. Gerhard Multhaup, McGill University
**W0-2**	Human Aβ	Mouse monoclonal	N-terminus of human Aβ (residues 4–10)	EMI Millipore, USA
**G2-10**	Human Aβ_40_	Mouse monoclonal	C-terminus of Aβ_40_	EMI Millipore, USA
**G2-13**	Human Aβ_42_	Mouse monoclonal	C-terminus of Aβ_42_	EMI Millipore, USA

### Bright-field immunohistochemistry

Free-floating immunostaining was done following well-established protocols [10, 11, 34, 35]. Sections were incubated in 0.3% hydrogen peroxide in PBS for 20 min, washed in PBS-T (0.01 M phosphate-buffered saline, 0.2% Triton X-100) and blocked 1 h with 10% normal goat serum (NGS) in PBS-T. To examine the evolution of the AD-like amyloid pathology we incubated sections with the monoclonal antibody McSA1 [36] (MediMabs, Montreal, Canada) at 1:4000 in PBS-T with 5% NGS overnight at 4°C. The following day, the sections were washed in PBS-T and incubated with a goat anti-mouse secondary antibody (MP Biochemicals, Canada) 1:100 in PBS with 5% NGS for 1 h. The sections were washed in PBS and incubated for 1 h with a mouse anti-peroxidase monoclonal antibody [37] (1:30) pre-incubated with horseradish peroxidase (5 μg/ml) in PBS (MAP kit, Medimabs, Canada). Stainings were developed with 0.06% 3,3′-diaminobenzidine (Sigma-Aldrich, USA) and 0.01% hydrogen peroxide (Sigma-Aldrich, USA) in PBS and then mounted on subbed slides. Sections were dehydrated in increasing ethanol concentrations (70-100%) and xylene, prior to coverslipping with Entellan (EM Science, USA). Images were acquired on an Axioplan Imaging microscope equipped with an AxioCam HRc digital camera (Carl Zeiss, Toronto, Canada); using the Axiovision 4.8 Software.

### Immunofluorescence and confocal microscopy

Immunofluorescence was done following established protocols [10, 38]. Sections were incubated in 50% ethanol for 20 min followed by three 10-min washes in PBS. Blocking was done in 10% NGS in PBS-T during 1 h. Primary antibodies were incubated overnight in 5% NGS at 4°C. Double labeling was performed between three anti-Aβ antibodies (McSA1, Medimabs, Canada; MOAB-2 [39], Biosensis, Australia and Nu1 [40], provided by Dr. William Klein, Northwestern University, USA; at 1:500) and a polyclonal antibody (pab27576, provided by Dr. Gerhard Multhaup, McGill University; at 1:500) directed against the last 43 amino acids of the C-terminal domain of APP. This antibody recognizes full-length APP (~100 kDa) and CTFs (~12 kDa) without crossreactivity for Aβ [13, 41, 42]. Alexa 488-conjugated goat anti-mouse (Jackson Immunoresearch, USA) and Alexa 594-conjugated goat anti-rabbit (Molecular Probes, USA) secondary antibodies were applied overnight at 1:400–1:800 in PBS with 5% NGS. Sections were washed three times in PBS and subsequently mounted on gelatin subbed slides, dried overnight at 4°C, and coverslipped with Aqua Polymount (Polysciences, USA).

Images were taken on a Zeiss LSM 510 confocal microscope (Carl Zeiss, Canada) equipped with Argon and Helium-Neon lasers. Three sections per animal were immunolabeled and five pictures per animal (per region) were acquired from area CA1 of the hippocampus and lamina V and III of the parietal cortex. To maximize the sensitivity of co-localization analysis and fully exploit the resolution of the microscope objective (Zeiss 63X oil immersion objective NA = 1.4), we used a 3.0 scan zoom so that each pixel covered 0.05 μm. Each signal was acquired using separate tracts with the appropriate laser-filter settings to avoid bleed-through of signals, and the pinhole was set so that optical sections were less than 0.7 μm thick. With the given objective settings, the theoretical resolution was of 250 nm in *x*, *y* and 700 nm in the *z* plane.

Confocal images were analyzed and quantified with the JACoP plugin [43] of the Image J software, to obtain Pearson and Manders’ correlation coefficients. The Pearson coefficient defines the quality of the linear relationship between two signals and varies between 1 and -1. Manders’ coefficients (M1 and M2) indicate the proportion of signal overlap; varying from 0 (no co-localization) to 1 (perfect co-localization). In this study, M1 indicated the proportion of McSA1 (or MOAB-2 or Nu1) (green signal) overlapping pab27576 immunoreactivity (red signal) over its total intensity. Conversely, M2 is defined as the proportion of pab27576 signal (red) overlapping McSA1/MOAB-2/Nu1 immunolabels (green) over its total intensity. Omission of primary antibodies resulted in no detectable fluorescent staining (data not shown).

### Structured Illumination Microscopy (SIM)

Given that both McSA1 and pab27576 gave robust fluorescent signals, we were able to image them by SIM a super-resolution microscopy technique permitting a resolution of ~100 nm in *x*, *y* and of ~300 nm in *z*[44]. The IHC was done following the same protocol as for the confocal experiments, except that the McSA1 and pab27576 were used at a concentration of 1:1000. Images were taken on a DeltaVision OMX V4 Blaze super-resolution microscope (Applied Precision, GE Healthcare). Reconstructed images were visualized using the Volocity 3D Image Analysis Software (PerkinElmer, USA). Following image reconstruction and registration, serial *z* planes were assembled to form a 3D model using the Volocity Software. A video showing different rotations and magnifications of the 3D model was made using the video tools from the software (Additional file 1: Video).

### Human Aβ_40_ and Aβ_42_ ELISA

Brains were homogenized in 8% vol (w/v) of cold TBS buffer (150 mM NaCl, 50 mM Tris / HCl, 5 mM EDTA, pH 7.6) containing a protease inhibitor cocktail (Roche, Germany) using a teflon-glass homogenizer. Homogenates were cleared by centrifugation at 100.000 g for 1 h at 4°C, and supernatants were collected for further analysis (TBS-soluble fraction). The remaining pellets were dissolved in 70% formic acid (FA) with 50% of homogenization volume and sonicated for 30 s at 30% power. Centrifugation at 100.000 g was repeated and supernatants were collected (FA-soluble fraction). Samples from FA-soluble fractions were neutralized with 1 M Tris, 0.5 M Na_2_HPO_4_ prior to ELISA analysis.

Levels of Aβ_40_ or Aβ_42_ in TBS-soluble and FA-soluble fractions were determined by sandwich ELISA. The C-terminus-specific anti-Aβ monoclonal antibodies G2-10 and G2-13 (EMI Millipore, USA) were used to capture Aβ species terminating at 40 or 42 amino acids, respectively. The monoclonal biotinylated W0-2 (EMI Millipore, USA) recognizing the Aβ N-terminus was used as detection antibody, and the reaction was developed by streptavidin-horseradish peroxidase conjugate and the chromogenic substrate 1-Step Ultra-TMB (Pierce, USA). After stopping the reaction with 1 M H_2_SO_4_, the enzymatic products were measured at 450 nm in a microplate reader (Anthos HT2, Germany). Synthetic Aβ_40_ or Aβ_42_ peptides (EMI Millipore, USA) served as standards.

### MALDI-MS analysis of Aβ peptides in rat CSF

Aβ was immunoprecipitated from 50 μl CSF with 5 μg of the anti-Aβ antibody W0-2 (EMI Millipore, USA) coupled to protein-G sepharose beads (GE Healthcare, USA) and incubated overnight at 4°C. The beads were washed three times with PBS and twice with 50 mM ammonium acetate, pH 7.0. Aβ was eluted twice with 50% acetic acid and vacuum-dried overnight. Immunoprecipitated samples were resuspended in 10 μl of TA (33% acetonitrile, 0.1% trifluoroacetic acid) and sonicated in a water bath for 10 min. Samples were spotted by the dried droplet technique mixing 1 μl of sample with 1 μl of a TA solution saturated with sinapinic acid. MALDI-MS analysis was carried out on a Bruker Ultraflex II instrument (Bruker Daltonik, Bremen, Germany).

### Behavioral studies

Tests were conducted during the light phase of the circadian cycle. Rats were handled prior to testing for 2–3 days. Experimenters were blinded to the genotype during all behavioral studies.

### Fear conditioning

The fear conditioning protocol was designed adapting a previously reported procedure [45]. Animals were tested in a single chamber connected to a weight transducer system to allow the tracking of movement (Panlab, Spain). The chamber was scented with coconut extract and cleaned with ethanol 70% between animals. To create a different environment for the fear memory retention test, the walls of the chamber were decorated with visual cues; the chamber was scented with mint extract and cleaned with acetic acid 1% between animals.

On day 1 (habituation) rats were allowed to explore the chamber for 5 min and returned to their home cages. On day 2 (conditioning), after an initial 90 sec phase of exploration (baseline), a 30 sec tone (75 dB, 5 kHz) was presented which co-terminated with a 2 sec foot shock (0.75 mA). Animals were allowed to recover (post-shock; 120 sec) and returned to their home cages. On day 3, contextual fear conditioning was evaluated by placing the animals in the chamber during 8 min. On day 4, animals were allowed to explore the “new” arena for 120 sec (baseline). This was followed by three consecutive tone presentations (30 sec, 75 dB, 5 kHz), each separated by a 30 sec pause. The Freezing software (Freezing v1.3.01, Panlab) recorded all freezing episodes in each of the test phases, considering freezing as immobility for at least 2 sec.

### Novel object recognition and location

Before testing, the animals were habituated to the testing environment by allowing them to explore the experimental arena. Following habituation, the rats were subjected to three testing phases: Exploration, Novel Object Location (NOL) and Novel Object Recognition (NOR). During exploration, five objects similar in size but different in color, shape and texture were put in the arena. These objects have been tested in pilot studies to ensure that none of them elicited spontaneous preference or avoidance. The animals were allowed to explore the objects for three sessions of 2 min, with a 10 min inter-session interval. An animal was considered as exploring when the muzzle was touching or in close proximity to the objects. The NOL test consisted of a single 2 min session where one of the objects was moved to a new location. For NOR, one of the undisplaced objects was substituted by a novel one. For NOL and NOR, the time exploring familiar and displaced/novel objects was recorded separately to calculate the discrimination ratio. We defined this ratio as the difference in exploration time for the displaced or novel object divided by total exploration time. Increased time spent exploring the object in the novel location was interpreted as successful spatial memory. Increased time spent exploring the novel object was interpreted as successful recognition memory for the familiar objects versus the novel one.

### Calculation of cognitive index

For correlation analysis between behavior and Aβ pathology, a global learning and memory score, referred as cognitive index, was calculated based on the animals’ performance during each different phase of behavior tests. For auditory and cue fear conditioning, a score was computed by calculating the fold increase in freezing behavior during the post-shock phase and tone presentation (respectively), compared to baseline freezing. Context conditioning freezing percentages were converted to a 10-point scale. For NOL and NOR, a score was obtained by calculating the fold increase between the animal’s discrimination ratio and that of chance levels. All behavior scores were added and expressed as a *z* score, referred here as “cognitive index”.

### Von Frey test

Animals were habituated to the testing environment by placing them in individual boxes on a metal mesh floor during 10 min. The following day mechanical sensitivity was assessed by measuring withdrawal responses to a series of calibrated Von Frey filaments with incrementing force (15 g, 26 g, 60 g and 100 g). These were applied perpendicularly to the plantar surface of both hind paws, following the “up and down” method [46]. Each hind paw was poked twice, calculating an average of all four pokes for between-animal comparisons.

### Statistical analysis

The software Graph Pad Prism 5.01 (La Jolla, CA, USA) was used for statistical analysis. One-way ANOVA was used for three-group comparisons, followed by post-hoc multiple comparison tests (as specified in figure legend). Two-way ANOVA was used to analyze the fear conditioning data. Graphs illustrate mean ± SEM.

## Results

### Progression of the amyloid pathology in McGill-R-Thy1-APP transgenic rats

This study provides a quantitative biochemical and morphological investigation of the evolution of the Aβ pathology and its associated cognitive impairments in McGill-R-Thy1-APP rats. In this rat model, the expression of Aβ-immunoreactive material is detectable as early as 1 week of age and the first isolated amyloid plaques may appear between 6 – 8 months starting in the subiculum [11].

**Figure 1:**
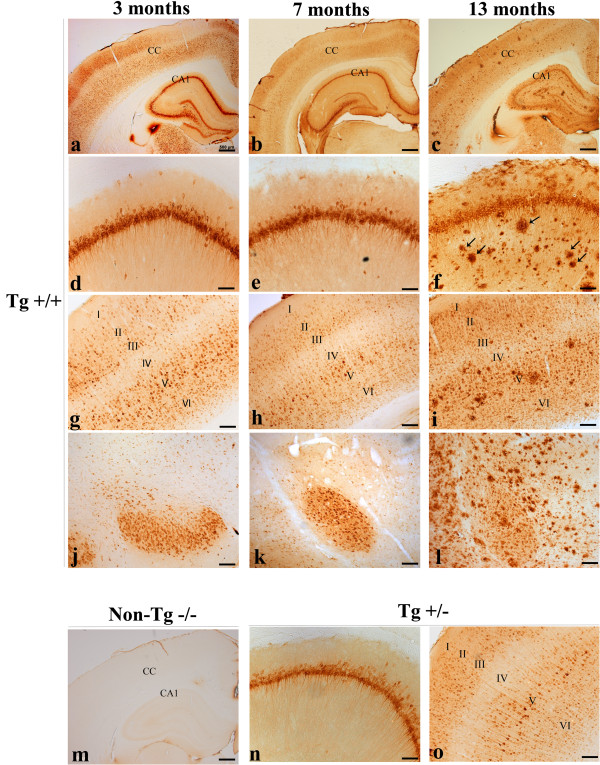
**Temporal progression of the AD-like amyloid pathology in McGill-R-Thy1-APP transgenic rats.** Intense intraneuronal Aβ immunoreactivity is observed in McGill transgenic rats throughout the neocortex **(a-c, g-i, o)**, hippocampus **(a-f, n)** and amygdala **(j-l)**, as detected with the anti-Aβ McSA1 monoclonal antibody. Note the absence of amyloid plaques at 3 months and 7 months of age. At 13 months, transgenic rats exhibited strong intracellular and extracellular McSA1 immunoreactivity. Abundant amyloid plaque deposition is evident throughout all cortical layers, hippocampal formation and amygdala in homozygote transgenic rats. Arrows indicate the presence of amyloid plaques. McSA1 immunoreactivity remained limited to the intracellular compartment in heterozygote animals. Note the absence of McSA1-immunoreactivity in non-transgenic animals **(m)**. Scale bar: a-c, m = 500 μm; d-l, n-o = 100 μm.

We defined the pre-plaque stage of the amyloid pathology as a stage where Aβ immunoreactivity is limited to the intraneuronal compartment in the absence of extracellular amyloid plaques, as detected with the anti-Aβ McSA1 monoclonal antibody (Figure 1). In this cohort, 3 and 7 month-old McGill transgenic rats displayed robust intracellular McSA1 immunoreactivity and no evidence of amyloid plaque deposition (Figure 1). By 13 months, homozygote (+/+) animals displayed widespread and abundant amyloid plaques throughout the cortex (with greatest expression in lamina V) (Figure 1c, i), hippocampus (Figure 1 c, f) and amygdala (Figure 1l). Heterozygote transgenic rats of comparable age (13 months) did not exhibit amyloid plaques (Figure 1n, o). In this rat model, the amyloid pathology at advanced stages (18–20 months) is further accompanied by strong microglial activation and dystrophic neuries surrounding amyloid plaques [11]. Absence of McSA1-immunoreactivity was observed in non-transgenic animals (Figure 1m), at all time points examined.

### Quantitative co-localization analysis between Aβ and APP/CTF-specific signals

To detect intracellular Aβ we utilized the monoclonal antibody (McSA1), which recognizes the N-terminal region of the human Aβ peptide (residues 1–12) [36]. This epitope could theoretically be found as well in β-CTF and in the APP molecule. However, competition studies have demonstrated that the McSA1 antibody is highly specific for Aβ as opposed to APP or sAPP-α [11, 36].

**Figure 2:**
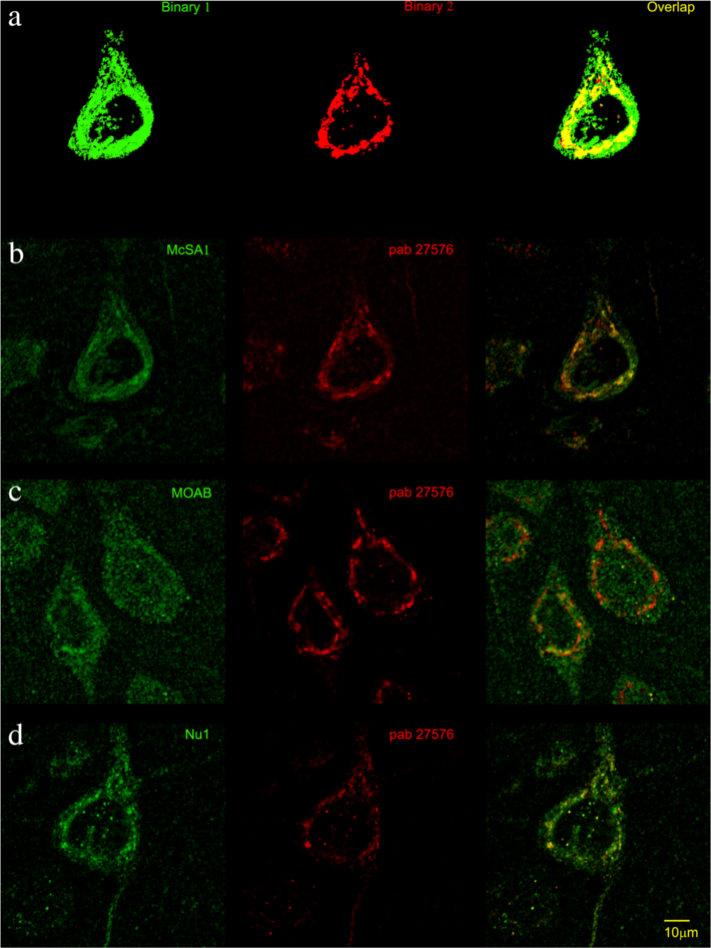
**Detection of intraneuronal Aβ and APP/CTFs. a)** To measure the overlap coefficient of different antibody binding sites, the signals were separated from background using a brightness criterion and made binary. The two images were then superimposed; the M1 coefficient represents the overlap (yellow) divided by the total green signal (yellow + green). The M2 coefficient represents the overlap divided by the total red signal (yellow + red). **b-d)** Representative high-magnification confocal micrographs depicting co-localization between pab27576 (red) and **b)** McSA1 (green) binding sites, **c)** MOAB-2 (green) and **d)** Nu1 (green) in lamina V neurons of the parietal cortex, at the pre-plaque stage (3 month-old transgenic rats). Note the lack of complete overlap between Aβ- and APP/CTF-specific signals. Scale bar = 10 μm.

We performed quantitative co-localization analysis between McSA1 and pab27576 immunoreactivities with the JACoP/Image J software (Figure 2a), at pre- and post-plaque stages of the amyloid pathology. At 3 months of age (pre-plaque stage) we observed only a partial co-localization between McSA1 and pab27576 immunoreactivity in neurons from lamina V (Figure 2b), as reflected by an average Pearson coefficient of 0.44 ± 0.03 (Table 2). Quantitative analysis demonstrated only ~30% overlap (M1 = 0.30 ± 0.03) between McSA1 and pab27576 signals in lamina V neurons (Table 2), indicating that ~70% of the cellular epitope recognized by McSA1 is not coming from APP or β-CTFs. Similar observations were made in CA1 neurons and in neurons from lamina III of the cortex (Additional file 2: Figure S1), revealing Pearson coefficients of 0.57 ± 0.02 and 0.32 ± 0.03 and a degree of overlap of only ~ 20 – 50% between the two signals (M1 = 0.48 ± 0.02 and M1 = 0.23 ± 0.03; respectively) (Table 2). The clear distinction between intracellular McSA1 and pab27576 immunoreactivities was also evident at later stages of the amyloid pathology (Additional file 2: Figure S1). Pearson coefficients ranged between 0.35 and 0.5 and there was approximately ~ 20 - 40% overlap between the two signals in the brain regions investigated (Table 2). At this time point, we also observed the presence of extracellular amyloid deposits that were McSA1 positive but pab27576 negative (Additional file 3: Figure S2c).

**Table 2:** **Co-localization analysis between McSA1 and pab27576**

		Pearson	Manders M1	Manders M2
	**CA1**	0.57 ± 0.02	0.48 ± 0.02	0.83 ± 0.02
3 months	**Lamina V**	0.44 ± 0.03	0.30 ± 0.03	0.65 ± 0.04
	**Lamina III**	0.32 ± 0.03	0.23 ± 0.03	0.56 ± 0.04
	**CA1**	0.51 ± 0.04	0.37 ± 0.02	0.79 ± 0.03
13 months	**Lamina V**	0.39 ± 0.03	0.22 ± 0.02	0.74 ± 0.03
	**Lamina III**	0.35 ± 0.03	0.22 ± 0.02	0.64 ± 0.04

M1: proportion of McSA1-IR (green) overlapping pab27576 (red) over its total intensity.

M2: proportion of pab27576 (red) overlapping McSA1-IR (green) over its total intensity.

**Figure 3:**
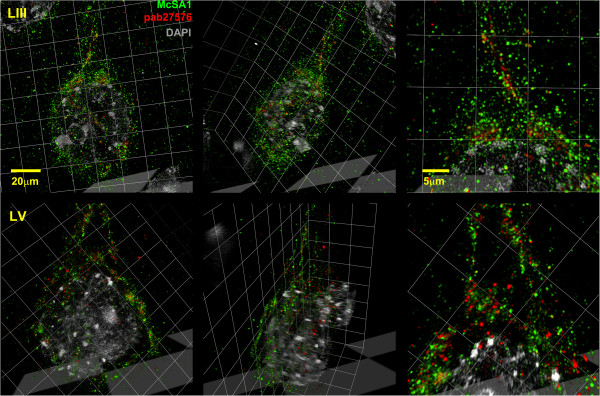
**Characterization of McSA1 and pab27576 immunoreactivities by super-resolution microscopy.** To further characterize the distribution of McSA1 and pab27576 signals, pyramidal neurons from lamina III and lamina V of the parietal cortex were imaged by SIM in 3-month old amyloid-plaque free transgenic rats. Note the distinct intracellular localization of McSA1 immunoreactivity (green) with that of pab27576 (red), denoting Aβ-amyloid peptides and APP/CTFs, respectively. DAPI (gray) indicates the boundaries of the neuronal nucleus. From left to right are frontal and oblique views, followed by higher magnification images of 3D-reconstructions.

Strikingly, the intracellular McSA1 immunolabeling appeared diffusely distributed throughout the neuronal cytoplasm, whereas the pab27576 signal was mostly associated to vesicular-like structures within the perinuclear region (Figure 2 and Additional file 2: Figure S1). To obtain a higher, optimal 3D resolution of McSA1 and pab27576 immunoreactivities at the pre-plaque stage, we applied super-resolution SIM microscopy. With this technique, we finally demonstrate that the overlap between McSA1 and pab27576 immunoreactive sites was minimal, where the former diffuse McSA1 signal now became localized to small intracellular vesicles, clearly dissociated from pab27576 immunoreactive structures (Figure 3). For a better illustration of the dissociation between Aβ and APP/CTF-specific signals, a 3D model was generated following image reconstruction and registration and assembly of serial *z* planes (Additional file 1: Video).

To further validate our inclusion/exclusion co-localization analysis we then stained rat brain sections with MAOB-2, a monoclonal antibody directed to residues 1–4 of Aβ, and which has been reported as absent of cross-reactivity with APP or CTFs [39]. In general, the MOAB-2 immunoreaction ranged from low (in some neurons) to hardly detectable in most neurons from the different areas and time points examined. Therefore, we subjected confocal images to quantitative co-localization analysis only when the MOAB-2 signal was strong enough to be clearly distinguished from background, non-specific staining. Corroborating our previous results with McSA1, we observed a differential pattern of immunoreactivity between MOAB-2 and pab27576 in cells with detectable and reliable MOAB-2 staining. At 3 months of age (pre-plaque) high-magnification quantitative co-localization analysis revealed only a partial overlap between the two signals in neurons from lamina V (Figure 2c), as reflected by an average Pearson coefficient of 0.41 ± 0.01 and M1 coefficient of 0.44 ± 0.02. At 13 months of age, the intracellular MOAB-2 immunolabel was hardly detectable in pyramidal neurons adjacent to amyloid plaques, the latter which were strongly labeled by this antibody and not by pab27576 (Additional file 3: Figure S2 a-b).

Additional confirmation of the segregation between Aβ-specific and APP/CTF-specific signals was obtained by co-labeling transgenic rat brain sections with the Nu1 monoclonal antibody (conformational, Aβ oligomer-specific) [40] and pab27576. With this approach, a similar clear distinction between Aβ-specific and APP/CTF-specific intraneuronal signals was observed. At the pre-plaque stage (3 months) we revealed only a partial co-localization between Nu1 and pab27576 immunoreactivity in neurons from lamina V (Figure 2d), indicated by an average Pearson coefficient of 0.53 ± 0.02 (Table 3). Quantitative analysis also demonstrated only ~40% overlap (M1 = 0.37 ± 0.02) between Nu1 and pab27576 signals in lamina V pyramidal neurons (Table 3). Similar observations were made in CA1 and lamina III (Additional file 4: Figure S3), revealing Pearson coefficients of 0.64 ± 0.01 and 0.35 ± 0.02 and a degree of overlap of ~ 25 – 50% between the two signals (M1 = 0.50 ± 0.02 and M1 = 0.24 ± 0.02; respectively) (Table 3). At 13 months of age co-localization analysis further revealed a clear distinction between intracellular Nu1 and pab27576 immunoreactivity (Additional file 4: Figure S3). Pearson coefficients ranged between 0.4 - 0.7 throughout the three brain regions and there was approximately 40 - 50% overlap between the signals (Table 3). At both stages of the amyloid pathology, the intracellular Nu1 immunolabel appeared associated to vesicle-like structures.

**Table 3:** **Co-localization analysis between Nu1 and pab27576**

		Pearson	Manders M1	Manders M2
	**CA1**	0.64 ± 0.01	0.50 ± 0.02	0.70 ± 0.02
3 months	**Lamina V**	0.53 ± 0.03	0.37 ± 0.02	0.61 ± 0.04
	**Lamina III**	0.35 ± 0.02	0.24 ± 0.02	0.43 ± 0.03
	**CA1**	0.63 ± 0.05	0.51 ± 0.03	0.84 ± 0.02
13 months	**Lamina V**	0.68 ± 0.04	0.49 ± 0.03	0.79 ± 0.03
	**Lamina III**	0.47 ± 0.02	0.37 ± 0.02	0.56 ± 0.03

M1: proportion of Nu1-IR (green) overlapping pab27576 (red) over its total intensity.

M2: proportion of pab27576 (red) overlapping Nu1-IR (green) over its total intensity.

### Analysis of Aβ peptides in brain

**Figure 4:**
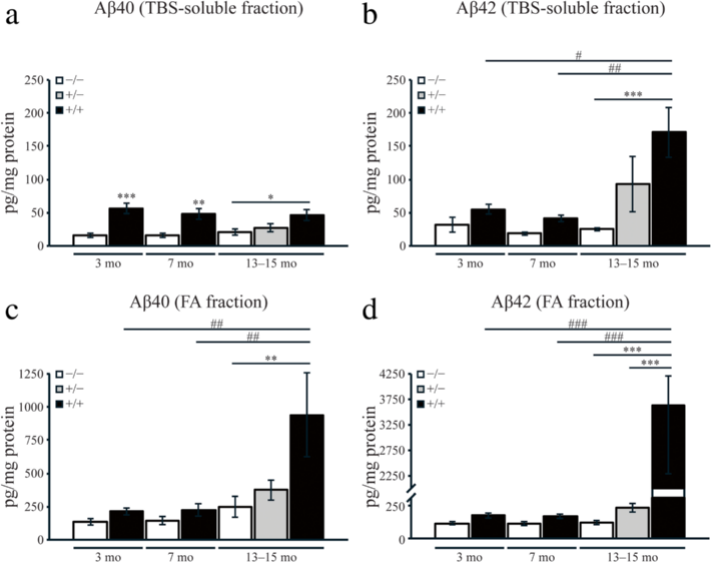
**Quantification of Aβ**_**40**_ **and Aβ**_**42**_ **levels in cortex and hippocampus by sandwich ELISA.** TBS-soluble **a)** Aβ_40_ and **b)** Aβ_42_ levels in cortex + hippocampus homogenates from non-transgenic (-/-), heterozygous (+/-) and homozygous transgenic (+/+) rats at different ages (3 months, 7 months and 13–15 months) were quantified with specific G2-10/W0-2 and G2-13/W0-2 sandwich ELISAs, respectively. TBS-insoluble pellets were extracted with 70% formic acid (FA) and after neutralization, **c)** Aβ_40_ and **d)** Aβ_42_ levels in FA fractions were assayed with G2-10/W0-2 and G2-13/W0-2 ELISAs, respectively. Values were normalized to total protein concentration and expressed as means ± SEM. One-way ANOVA, followed by Dunnett’s post-hoc test. *P < 0.01, **P < 0.001, ***P < 0.0001 (across the same time point), ^#^ *P* < 0.01, ^##^ *P* < 0.001, ^###^ *P* < 0.0001 (across ages).

We further quantified the age-dependent accumulation of Aβ peptides in McGill transgenic rat brain homogenates by ELISA following established protocols [47, 48]. We observed significantly higher levels of soluble Aβ_40_ peptides (~3 fold) in cortex and hippocampus from transgenic rats compared to age-matched non-transgenic animals, at 3 months (p < 0.0001), 7 months (p < 0.001) and 13–15 months of age (p < 0.01) (Figure 4a). The levels of soluble Aβ_40_ peptides, however, did not differ significantly between transgenic rats at different stages of the amyloid pathology, with an average of 50.2 ± 4.7 pg/mg total protein. Furthermore, cortical and hippocampal levels of TBS-soluble Aβ_42_ peptides were slightly elevated in young McGill transgenic rats (3 and 7 months) compared to non-transgenic animals (Figure 4b), whereas substantially more Aβ_42_ was found in 13–15 month-old transgenics (171 ± 37.8 pg/mg protein, p < 0.0001). This data strongly suggests an age-dependent accumulation of soluble Aβ_42_ peptides in McGill transgenic rats (p < 0.001). Notably, levels of TBS-soluble Aβ_40_ and Aβ_42_ were slightly increased in heterozygote (+/-) rats at 13–15 months of age compared to non-transgenic littermates, though not significantly (Figure 4a-b).

When analyzing the presence and levels of Aβ in FA fractions we detected highest concentrations of Aβ_40_ (938.3 ± 313.9 pg/mg protein, p < 0.001) and Aβ_42_ (3246.1 ± 963.7 pg/mg protein, p < 0.0001) in transgenic rats at 13–15 months of age compared to plaque-free transgenic rats (Figure 4c-d), with an average Aβ_42_/Aβ_40_ ratio of 6 ± 2. Thus, this data revealed that ~95% of the total Aβ_40_ and Aβ_42_ detected in the cortex and hippocampus of transgenic rat at the post-plaque stage required FA for solubilization. There was no difference or age-dependent increase in the level of TBS-soluble or FA-treated Aβ peptides in cerebellum between non-transgenic and transgenic animals (Additional file 5: Figure S4); a finding that is in agreement with the human pathology [49].

### Analysis of Aβ peptides in CSF

**Figure 5:**
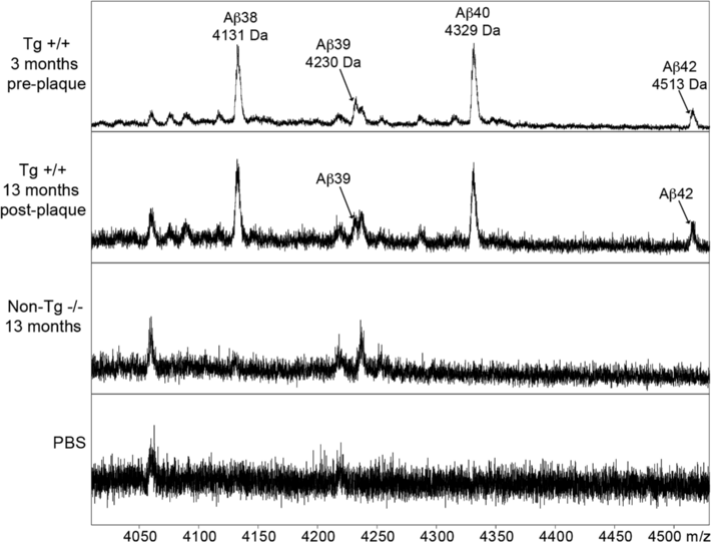
**MALDI-MS spectra of immunoprecipitated Aβ peptides from rat CSF.** Aβ peptides were immunoprecipitated from rat CSF with the W0-2 antibody and further subjected to MALDI-MS analysis. Peaks correspond to signals for Aβ_38_, Aβ_39_, Aβ_40_ and Aβ_42_ with theoretical monoisotopic masses of 4130.0 Da, 4229.0 Da, 4328.2 Da and 4512.3 Da respectively. Note that the differences in measured versus theoretical masses are predominantly due to the linear detection method used. Signals observed in the non-transgenic animals and the PBS control result from either protein-G sepharose, the antibody or other non-specific binding components of the CSF.

In order to detect Aβ peptides in CSF, we immunoprecipitated human Aβ with the W0-2 antibody (Table 1). MALDI-MS analysis revealed the presence of Aβ_42_ (4513 Da), Aβ_40_ (4329 Da), Aβ_39_ (4230 Da) and Aβ_38_ (4131 Da) peptides in CSF from McGill transgenic rats, both at pre- and post-plaque stages of the amyloid pathology (Figure 5).

Notably, the pattern of CSF peptides was similar at both time points and comparable to that observed in human CSF [50]; except for two observations: no Aβ_37_ was detected in the CSF of McGill transgenic rats at any time point and the Aβ_38_ signal was as strong as the Aβ_40_ signal, under these conditions. MALDI-MS analysis revealed an absence of Aβ signal in non-transgenic animals (Figure 5) at both time points, indicating that detected Aβ peptides predominantly derive from the human APP transgene expression or reflecting the low level of endogenous rodent Aβ [51]. The masses identified as Aβ peptides were absent when immunoprecipitation was performed from PBS instead of CSF.

### Early and progressive cognitive deficits in McGill-R-Thy1-APP rats

#### Impaired fear conditioning

**Figure 6:**
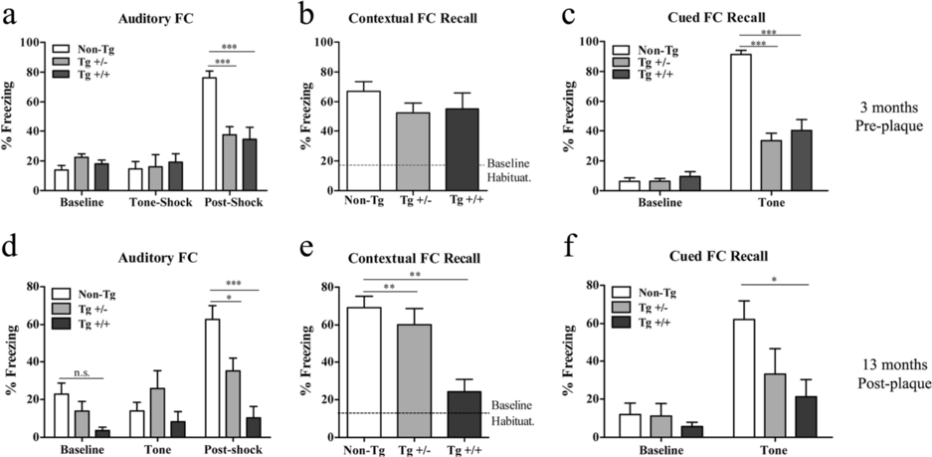
**Impaired auditory fear conditioning in McGill-Thy1-APP transgenic rats.** Freezing responses in **a-c)** 3 month-old and d-f) 13 month-old transgenic rats. Following two days of habituation and animal handling, auditory fear conditioning was tested **(a, d)** followed by assessment of contextual fear conditioning 24 h later **(b, e)**. The following day (48 hs after auditory conditioning), cued fear responses were examined in a different arena **(c, f)**. Note the progression of auditory and contextual fear conditioning deficits as the amyloid pathology advanced from pre- to post-plaque stages. **a, c, d, f)** Two-Way ANOVA, followed by Bonferroni post-hoc test. **b, e)** One-Way ANOVA, followed by Bonferroni post-hoc test. Error bars represent mean ± SEM. *P < 0.05, **P < 0.01, ***P < 0.001.

During auditory conditioning training (Figure 6a), 3 month-old transgenic and non-transgenic animals exhibited low, comparable baseline freezing behavior. The initial presentation of the tone, before the foot-shock, did not result in increased freezing behavior in any of the groups. Conversely, we observed marked differences in fear response acquisition between transgenic animals and non-transgenic littermates during the post-shock phase (F_2,21_ = 17.03; p < 0.001).

In the fear conditioning paradigm, animals can also learn to associate the environment with the aversive stimulus; therefore, we tested contextual fear conditioning behavior 24 h later in the same arena, and compared it to the average freezing response during the pre-training habituation session (which averaged ~10% freezing). No differences in freezing responses between non-transgenic and transgenic rats were detected during contextual fear conditioning testing at this early stage (F_2,21_ = 1.043; p > 0.05) (Figure 6b). Following contextual fear conditioning, we then tested fear memory retrieval in response to an auditory cue, within a different environment. All rats exhibited low, comparable freezing responses at baseline. However, upon tone presentation, McGill transgenic rats manifested significant deficits in amygdala-dependent auditory fear memory, compared to non-transgenic littermates (F_2_,_21_ = 47.12; p < 0.001) (Figure 6c). Therefore, fear conditioning deficits were detectable at early stages of the amyloid pathology, in the absence of amyloid plaques. At this early time-point, transgenic homozygotes and heterozygote rats had comparable fear conditioning responses throughout all test phases. However, with increasing age and advancing amyloid pathology, fear conditioning deficits severely progressed in homozygote animals (Figure 6d-f).

We observed marked differences in fear response acquisition between transgenic and non-transgenic rats during the post-shock phase at 13 months (Figure 6d). Both heterozygote and homozygote rats exhibited lower freezing responses compared to non-transgenic animals (F_2,19_ = 13.59; p < 0.05 and p < 0.001). Non-transgenic animals presented a significant increase in freezing behavior, with respect to their baseline response (F_2,14_ = 25.6; p < 0.001). There was a mild learning response in heterozygote animals (F_2,14_ = 4.95; p < 0.05) with respect to their baseline freezing behavior. Conversely, transgenic homozygote rats exhibited significant fear conditioning deficits as evidenced by comparable freezing levels throughout all test phases (F_2,10_ = 1.01; p > 0.05). All animals exhibited low, comparable freezing behavior at baseline and during the initial presentation of the tone.

In 13-month old transgenic rats with advanced amyloid pathology we revealed marked contextual fear conditioning deficits in homozygote animals. Non-transgenic and transgenic heterozygote rats exhibited high freezing responses (~70% and ~60% freezing, respectively) (Figure 6e). Conversely, freezing behavior was significantly lower in McGill homozygote transgenic rats (F_2_,_19_ = 9.528; p < 0.01). During fear memory retrieval in a different context (Figure 6f), all groups exhibited low, comparable baseline freezing. Upon tone presentation, homozygote rats presented significantly lower freezing responses compared to non-transgenic animals (F_2,19_ = 3.37; p < 0.05).

Importantly, at both time points examined, there were no differences in locomotor activity and/or pain sensitivity between rat groups (Additional file 6: Figure S5), suggesting that the reduced fear conditioning responses (freezing behavior) in transgenic rats were not influenced by any differences in sensitivity to pain or by increased locomotion.

#### Impaired novel object recognition & location

**Figure 7:**
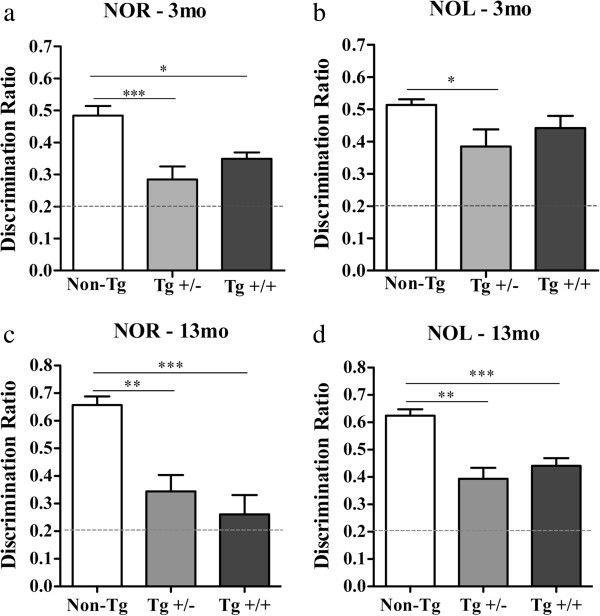
**Deficits in Novel Object Recognition and Location task.** In the novel object recognition test (NOR) the object discrimination ratio was defined as the difference in exploration time for the novel object (in sec) divided by the total exploration time (in sec). **a, c)** McGill APP transgenic rats exhibited lower recognition memory indices than wild type littermates at **a)** pre-plaque (3 months) and **c)** post-plaque (13 months) stages. In the novel object location test (NOL) **(b, d)** the object discrimination ratio was defined as the difference in exploration time for the displaced object (sec) divided by the total exploration time (in sec). Data is expressed as mean ± SEM and analyzed with One-Way ANOVA, followed by Tukey’s post hoc test; *P < 0.05, **P < 0.01, ***P < 0.001.

In the novel object recognition test, McGill-R-Thy1-APP rats were able to discriminate the novel object better than chance levels (object recognition index = 0.2), however they exhibited significantly lower object recognition indices compared to wild type animals (Figure 7). An impairment in object recognition memory was detectable at early stages of the amyloid pathology, months prior to extracellular Aβ deposition (Figure 7a) in McGill transgenic rats (F_2_,_19_ = 10.61; p < 0.001 and p < 0.05, respectively). Novel object location (Figure 7b) was also significantly impaired in heterozygote animals at this early stage but, despite the trend, did not appear significantly affected in homozygote rats (F_2_,_19_ = 3.031; p < 0.05 and p > 0.05, respectively). At 13 months of age, McGill transgenic heterozygote and homozygote rats showed significant impairments in object recognition (Figure 7c, F_2,21_ = 13.87; p < 0.001), with recognition indices close to chance levels, as well as deficits in location memory (Figure 7d, F_2,21_ = 16.41; p < 0.001), compared to non-transgenic animals.

## Discussion

The concept that Aβ accumulates within neurons remains a matter of controversial debate. However, it is widely accepted that a central event in AD pathogenesis is the abnormal accumulation of Aβ and its related toxins, either due to increased production or to deficits in clearance mechanisms [52, 53]. Aβ peptides, initially discovered extracellularly, are the core component of amyloid plaques in AD and DS brains [54, 55]. Several studies have reported the existence of intracellular Aβ within cultured neurons [36, 56–59] as well as in post-mortem brains from AD [14, 16, 60], DS subjects [17–19] and in transgenic rodent models [10, 12, 13, 61, 62].

Despite the above-mentioned studies, a valid concern has been raised regarding some of the antibodies applied for intracellular Aβ immunodetection, which could theoretically also recognize the Aβ sequence as part of APP and in CTFs. This argument was recently put forward in a study by Winton and colleagues who further concluded that the intraneuronal material in the well-established 3xTg-AD mouse model is not Aβ but in fact, solely APP [30]. The idea that only APP accumulates intraneuronally is thought-provoking and additionally, whether the intracellular Aβ pathology causes some of the symptoms in human AD remains unknown.

In this report, we have investigated the molecular species that accumulate intraneuronally in McGill-R-Thy1-APP rats and examined in parallel the presence of cognitive impairments, in a model which is closer to the human pathology than transgenic mice. To investigate the intracellular Aβ pathology, we have performed high-magnification quantitative co-localization/exclusion analysis, using three different monoclonal antibodies specific for Aβ: 1) McSA1, against Aβ residues 1–12; 2) MOAB-2, against Aβ residues 1–4 and 3) Nu1, a conformation-specific antibody which recognizes Aβ oligomers [40]. These antibodies were co-incubated with a polyclonal antibody (pab27576) against the C-terminal domain of full-length APP. The present study revealed a consistent subcellular segregation of immunoreactive sites detected by Aβ-specific antibodies and pab27576 within neurons of different brain areas, at pre- and post-plaque stages of the amyloid pathology. Importantly, our co-localization/exclusion analysis also revealed that the intracellularly accumulated material is likely a heterogeneous mixture of Aβ, APP and CTFs.

The examination of the amyloid pathology with super-resolution microscopy permitted us to further reveal a clear separation of the subcellular location of Aβ-immunoreactivity from that of APP/CTFs. Similar compelling evidence supporting the existence of *bonafide* intraneuronal Aβ peptides has been provided by ELISA analysis on laser microdissected pyramidal neurons from sporadic AD cases [63]. Future studies of this nature are warranted in the McGill-R-Thy1-APP model.

We have observed a certain degree of overlap between Aβ antibodies and pab27576 by confocal microscopy. This likely reflects the presence of Aβ and APP/CTFs within the same intracellular location but does not necessarily imply that the antibodies are identifying the same molecules. In the experimental conditions employed for the confocal imaging, the resolution was of 250 nm in the *x*, *y* planes, and 700 nm in *z*; an area sufficient to accommodate thousands of molecules and even neighboring organelles. Notably, when the McSA1 and pab27576 signals were resolved by SIM, McSA1 immunoreactivity became almost completely dissociated from pab27576 and was found in distinct compartments. It should be kept in mind, however, that this super-resolution technique also has its limitations. Because the mathematical algorithms reconstruct images based on Moiré patterns caused by the structured illumination, the technique is less effective at reassigning signal that is diffuse or weak. It is then possible that we have failed to detect low levels of Aβ or APP at plasma membrane locations.

Previous work on the McGill-R-Thy1-APP transgenic rat has provided additional evidence to support that a considerable component of the intracellular material detected by McSA1 is Aβ. Pre-adsorption of this antibody with synthetic Aβ_42_ completely abolished McSA1 immunoreactivity. Conversely, incubation with the same molar concentration of a cleaved fragment of APP did not result in diminished signal intensity [11], reinforcing the specificity of the McSA1 antibody for Aβ compared to APP-derived peptides [36].

In this study, we further observed that the intraneuronal Aβ-specific immunolabel was hardly detectable in close proximity to amyloid deposits. One interpretation could be that the intracellular pool of Aβ may act as a source for the formation of extracellular amyloid deposits [14]. Notably, in DS brains intracellular Aβ immunoreactivity diminishes with increasing age and advanced AD pathology [18, 19]. Furthermore, in brains from APP transgenic mice conformation-specific antibodies have revealed the early appearance of intraneuronal fibrillar and oligomeric Aβ immunoreactivity, which declined as amyloid plaques appeared, and further became evident in the extracellular space [10]. Alternatively, the apparent decline in intracellular Aβ signal may be the result of the intense reactivity of extracellular Aβ, potentially sequestering antibodies and creating steric hindrance, thus lowering the availability of intracellular epitopes.

The biochemical data presented in this report indicates that soluble Aβ_40_ and Aβ_42_ peptides are already abundant during amyloid plaque-free stages. We assert that this material reflects true Aβ (cleaved from APP), as the ELISA approach applied in this study consisted of C-terminus specific anti-Aβ capture antibodies, which do not recognize CTFs or APP molecules. In the McGill transgenic rat, ELISA analysis revealed robust accumulation of soluble Aβ species at 3 and 7 months of age (in cortex and hippocampus), with a preferential progressive and incremental accumulation of Aβ_42_ compared to Aβ_40_ peptides at advanced ages. Such a situation is similar to that observed in human AD and DS brains [16, 18, 64]. This finding is of great significance considering that cognitive deficits paralleled the early accumulation of soluble Aβ peptides and further progressed across age and with the advancement of the soluble Aβ_42_ pathology.

**Figure 8:**
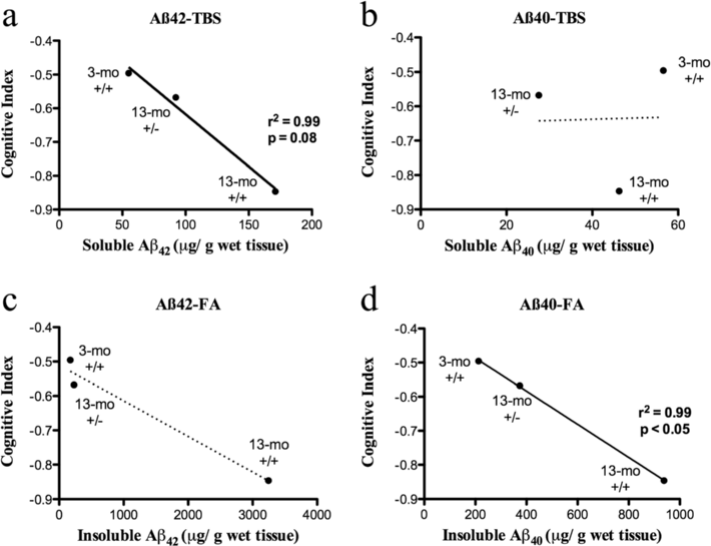
**Link between soluble Aβ pathology and cognitive impairment.** Correlation between average levels of soluble **(a, b)** and insoluble **(c, d)** Aβ_42_ and Aβ_40_ and the magnitude of cognitive impairments in transgenic animals, during the temporal progression of the AD-like amyloid pathology. A cognitive index was calculated based on the animals’ performance throughout all phases of the two behavioral paradigms employed, and expressed as a *z* score. Average values were analyzed with linear regression. Note the negative correlation between cognition and the levels of soluble Aβ_42_ peptides but not with soluble Aβ_40_.

To further address this issue, we analyzed whether there was an association between the average concentrations of Aβ peptides and the magnitude of behavior deficits, across the temporal progression of the amyloid pathology in transgenic animals. We calculated a cognitive index, adding the scores obtained throughout the different phases of the two behavioral paradigms employed, and expressing them as a *z* score. This analysis revealed a trend reflecting that higher concentrations of soluble Aβ_42_ peptides correlated with lower indices of cognitive performance (Figure 8a). No such correlation was evident for soluble Aβ_40_ peptides (Figure 8b) or insoluble Aβ_42_ (Figure 8c). However, there was a significant association between the levels of insoluble Aβ_40_ peptides and cognitive decline (Figure 8d). Although this correlation analysis cannot establish causality it is consistent with the concept that soluble forms of Aβ_42_ are highly toxic species.

To determine the occurrence of Aβ peptides in the rat CSF we resorted to Aβ immunoprecipiation followed by MALDI-MS analysis, before and after the formation of amyloid deposits. This technique allowed the detection of C-terminally truncated peptides, including Aβ_38_, Aβ_39_, Aβ_40_ and Aβ_42_ species, as early as 3 months of age; a time point which precedes amyloid plaque deposition by several months (4–6 months). The distinct pattern of Aβ peptides observed in rat CSF compared to human CSF (which includes more species, ranging from 1–12 to 1–42) may reflect differences in the γ-secretase complex or differential Aβ clearance patterns between human and rodent species. Importantly, this is one of the first reports applying Aβ immunoprecipitation followed by MALDI-MS analysis in CSF from a transgenic AD model, providing a robust, novel platform for the assessment of Aβ expression profiles in biological fluids of transgenic models. Furthermore, the CSF detection of Aβ peptides suggest a dynamic state of these molecules, where some accumulate inside neurons, and some are exported to the extracellular space, from where they can reach the CSF even in the absence of amyloid plaques in the brain parenchyma.

A good number of previous studies support the tenet that the occurrence of intraneuronal Aβ accumulation can have early, deleterious effects in CNS functions [8, 12, 61, 65]. In our study, we observed strong Aβ-immunoreactivity in the amygdala at pre-plaque (intraneuronal) and post-plaque stages; changes which likely account for the fear conditioning deficits reported at both time points. Emotional disturbances are a common early symptom in AD patients and such changes may be a reflection of early amygdalar damage [66, 67]. The fact that cognitive impairments were seen at the pre-plaque stage, where Aβ could be detected intraneuronally (by IHC) and in CSF (by MALDI-MS), suggests that a combination of intraneuronal and soluble extracellular Aβ may be responsible for impairing neuronal function at early time points. A role for Aβ-mediated toxicity is further supported by the fact that cognitive deficits progressed according with the incremental accumulation of the amyloid pathology, in particular, increased levels of soluble Aβ_42_ (Figure 8a). However, we do not discard the possibility that the presence of other intracellular species, such as CTFs may also contribute to neuronal dysfunction in McGill transgenic rats. β-CTF can be toxic to primary rat hippocampal neurons in culture [68], and is capable of inducing impairments in spatial learning and working memory *in vivo*[69–71].

Finally, we propose that an analogous, intraneuronal Aβ pathology during the pre-diagnostic stages of human AD should also have a negative impact on cognitive function. However, due to population-based tests and the well-known aspect of neural reserve [72] such early deficits may be difficult to reveal in the human species. We therefore suggest that self-to-self assessments that reflect a cognitive decline from an individual-adjusted baseline may be required to detect early (and yet subtle) memory deficits in pre-symptomatic human AD.

## Conclusions

This study demonstrates that a considerable component of the intracellular AD-like pathology in McGill-Thy1-APP rats consists of Aβ peptides. The intracellular material reflects a variety of molecular species, including free Aβ, APP, CTFs as well as aggregated Aβ peptides in the form of oligomers. Future investigations on this issue are warranted, particularly to examine which form of Aβ predominates within neurons and in which compartment; as well as dissecting the differential contributions of intraneuronal species to the development and progression of CNS dysfunction. A similar pathological situation is likely to occur at the earliest, silent stages of AD progression in the human brain.

## Electronic supplementary material

Additional file 1: **Video.** Following image reconstruction and registration, serial *z* planes were assembled to form a 3D model using the Volocity 3D Image Analysis Software. The video shows different rotations and magnifications of the 3D model revealing a clear dissociation between intraneuronal McSA1 (green) and pab27576 (red) immunoreactivities. (MOV 19 MB)

Additional file 2: Figure S1: Double immunolabeling with McSA1 and pab27576 antibodies. Representative high-magnification confocal micrographs depicting lack of complete co-localization between pab27576 (red) and McSA1 (green) immunoreactive sites at 3 months and 13 months in CA1 neurons of the hippocampus, and neurons of lamina V and III of the parietal cortex. Note the lack of complete overlap between the intracellular Aβ- and APP/CTF-specific immunoreactive signals at both time points. Scale bar = 10 μm. (TIFF 28 MB)

Additional file 3: Figure S2: Double immunolabeling with MOAB-2, McSA1 and pab27576. a-b) Representative high-magnification confocal micrographs depicting co-localization between pab27576 (red) and MOAB-2 (green) in CA1 neurons of the hippocampus a) and neurons of lamina V of the parietal cortex b) at post-plaque stages (13 months). c) Co-localization between McSA1 (green) and pab27576 (red) immunoreactive sites in neurons of the cerebral cortex (lamina V) at 13 months. Note the absence of pab27576 immunoreactivity in amyloid plaques (a-c). Scale bar = 10 μm. (TIFF 20 MB)

Additional file 4: Figure S3: Double immunolabeling with Nu1 and pab27576. Representative high-magnification confocal micrographs depicting lack of complete co-localization between pab27576 (APP/CTF-specific sites, red) and Nu1 (Aβ oligomer-specific sites, green). These images illustrate CA1 neurons of the hippocampus and neurons of lamina V and III of the parietal cortex from animals aged 3 and 13 months. Scale bar = 10 μm. (TIFF 30 MB)

Additional file 5: Figure S4: Quantification of Aβ_40_ and Aβ_42_ levels by ELISA in cerebellum. Aβ_40_ and Aβ_42_ levels in cerebellum homogenates from non-transgenic (-/-), heterozygous (+/-) and homozygous transgenic (+/+) rats at different ages (3 months, 7 months and 13–15 months) were quantified with specific G2-10/W0-2 and G2-13/W0-2 sandwich ELISAs, respectively. Values were normalized to total protein concentration and expressed as means ± SEM. One-way ANOVA, followed by Dunnett’s post-hoc test. (TIFF 23 MB)

Additional file 6: Figure S5: Analysis of locomotor activity and pain sensitivity in McGill transgenic rats. a, c) Freezing responses recorded during a 5 min exploratory session in the testing environment. The animals were allowed to explore the arena and returned to their home cages. No stimuli were presented. b, d) Analysis of tactile sensitivity in the rat hind paw using Von Frey filaments of increasing force. The graph depicts the average withdrawal thresholds to mechanical stimulation, expressed in grams of pressure. Data is expressed as mean ± SEM. One-Way ANOVA, followed by Bonferroni post-hoc tests. (TIFF 3 MB)

## Abbreviations

AD: Alzheimer’s disease
Aβ: Amyloid-β
APP: Amyloid precursor protein
CSF: Cerebro-spinal fluid
CTF: C-terminal fragment
DS: Down syndrome
FA: Formic acid
IHC: Immunohistochemistry
MALDI: Matrix assisted laser desorption ionization
MS: Mass spectrometry
NGS: Normal goat serum
NOL and NOR: Novel object location and recognition
PBS: Phosphate-buffered saline
sAPP-α: soluble APP-alpha
SIM: Structured illumination microscopy
TBS: Tris-buffered saline.
