# Supplementary figures and images for: Intracellular Aβ pathology and early cognitive impairments in a transgenic rat overexpressing human amyloid precursor protein: a multidimensional study
Source: Acta Neuropathol Commun. 2014 Jun 5;2:61. doi: 10.1186/2051-5960-2-61 (PMC4229908; doi:10.1186/2051-5960-2-61)

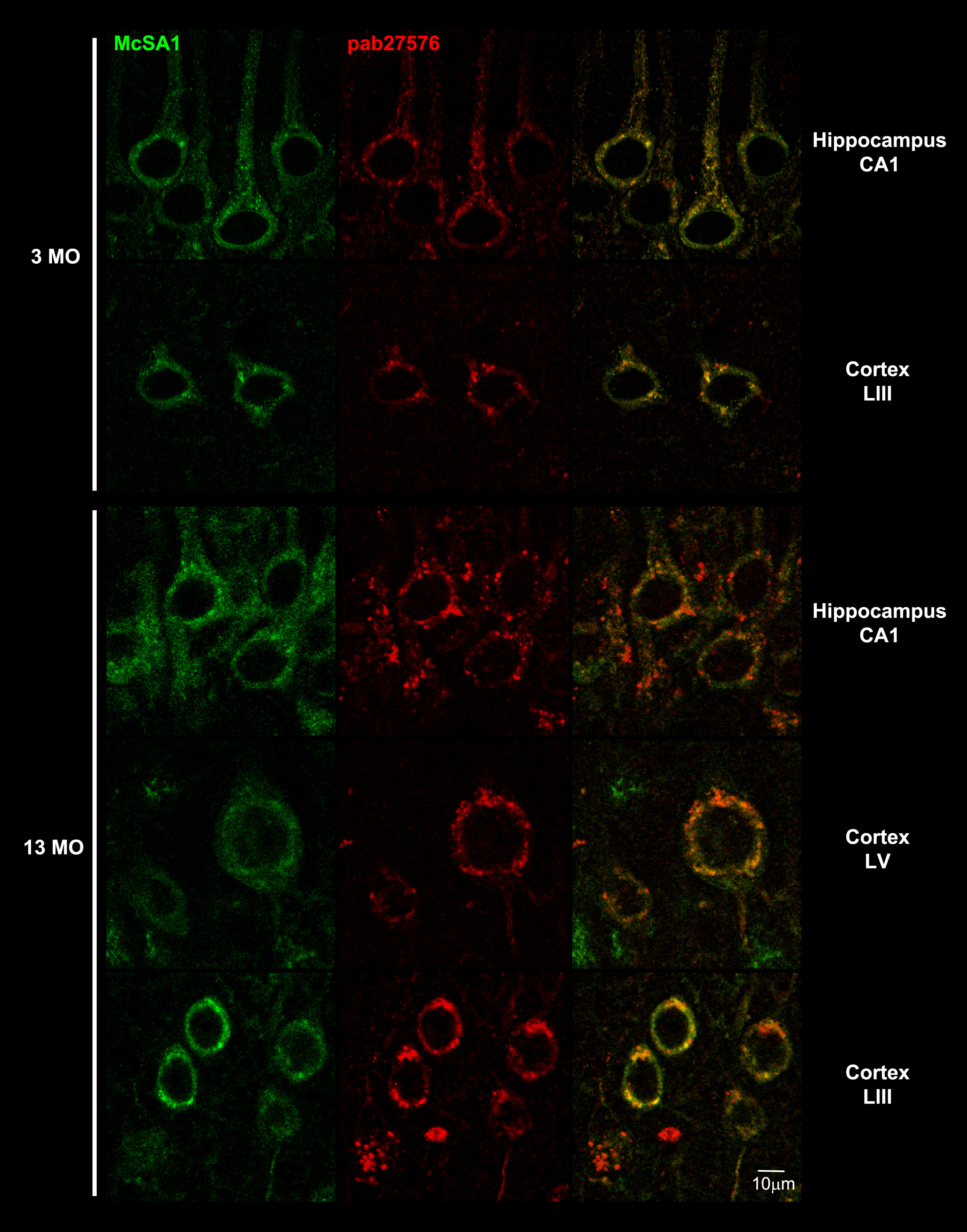

Supplement: Supplementary file 2 — Additional file 2: Figure S1: Double immunolabeling with McSA1 and pab27576 antibodies. Representative high-magnification confocal micrographs depicting lack of complete co-localization between pab27576 (red) and McSA1 (green) immunoreactive sites at 3 months and 13 months in CA1 neurons of the hippocampus, and neurons of lamina V and III of the parietal cortex. Note the lack of complete overlap between the intracellular Aβ- and APP/CTF-specific immunoreactive signals at both time points. Scale bar = 10 μm. (TIFF 28 MB) [file 40478_2014_135_MOESM2_ESM.tiff]

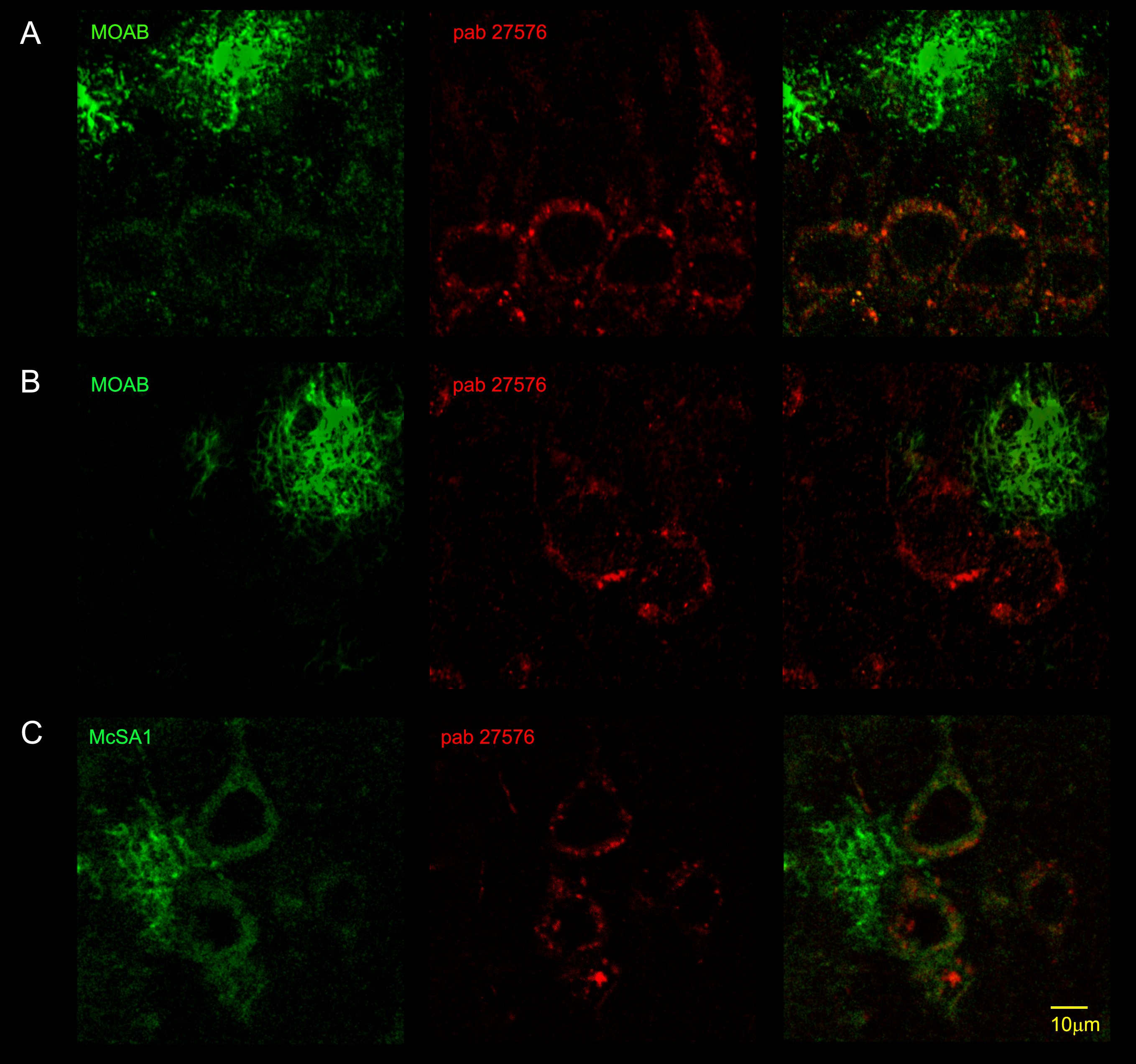

Supplement: Supplementary file 3 — Additional file 3: Figure S2: Double immunolabeling with MOAB-2, McSA1 and pab27576. a-b) Representative high-magnification confocal micrographs depicting co-localization between pab27576 (red) and MOAB-2 (green) in CA1 neurons of the hippocampus a) and neurons of lamina V of the parietal cortex b) at post-plaque stages (13 months). c) Co-localization between McSA1 (green) and pab27576 (red) immunoreactive sites in neurons of the cerebral cortex (lamina V) at 13 months. Note the absence of pab27576 immunoreactivity in amyloid plaques (a-c). Scale bar = 10 μm. (TIFF 20 MB) [file 40478_2014_135_MOESM3_ESM.tiff]

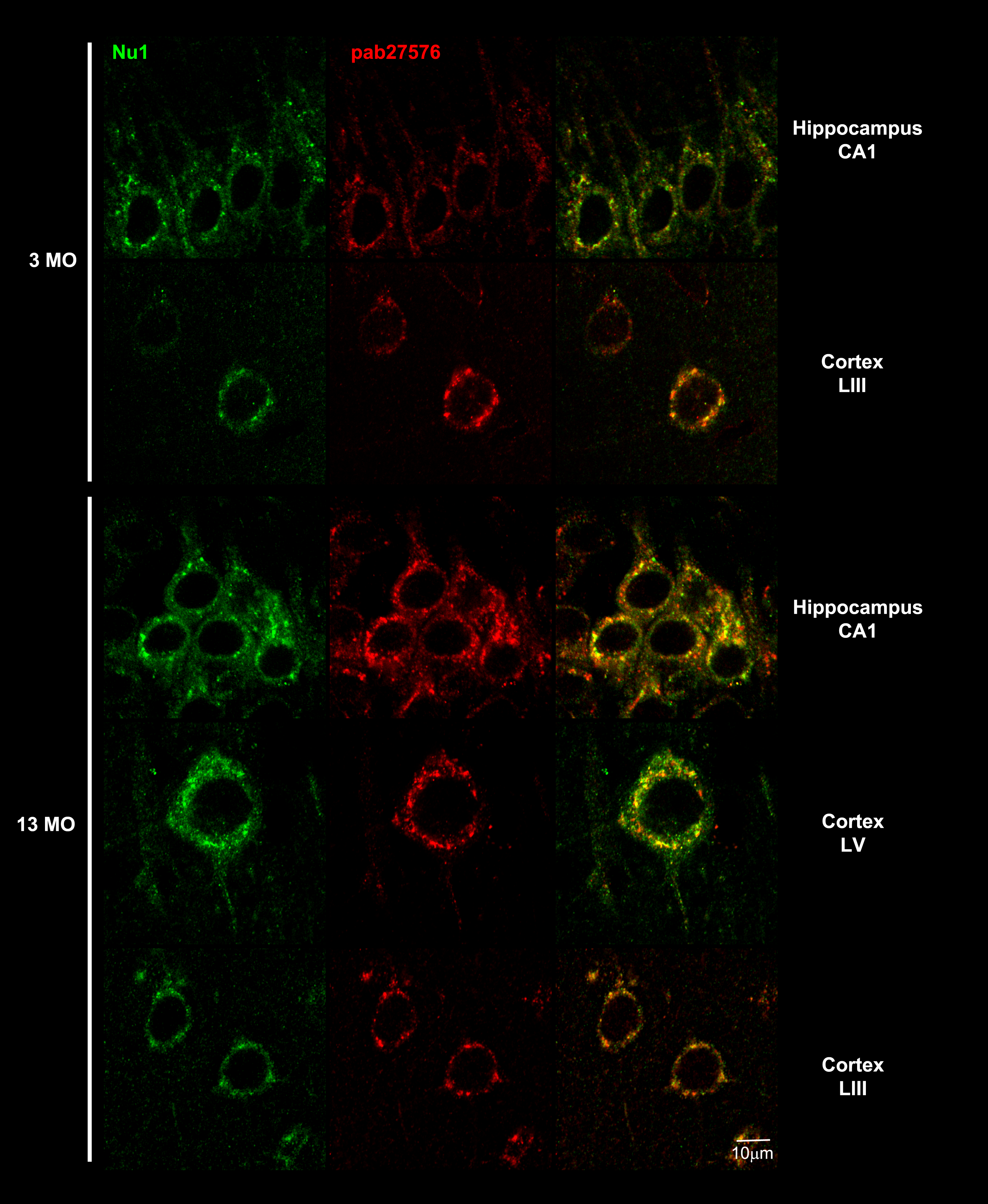

Supplement: Supplementary file 4 — Additional file 4: Figure S3: Double immunolabeling with Nu1 and pab27576. Representative high-magnification confocal micrographs depicting lack of complete co-localization between pab27576 (APP/CTF-specific sites, red) and Nu1 (Aβ oligomer-specific sites, green). These images illustrate CA1 neurons of the hippocampus and neurons of lamina V and III of the parietal cortex from animals aged 3 and 13 months. Scale bar = 10 μm. (TIFF 30 MB) [file 40478_2014_135_MOESM4_ESM.tiff]

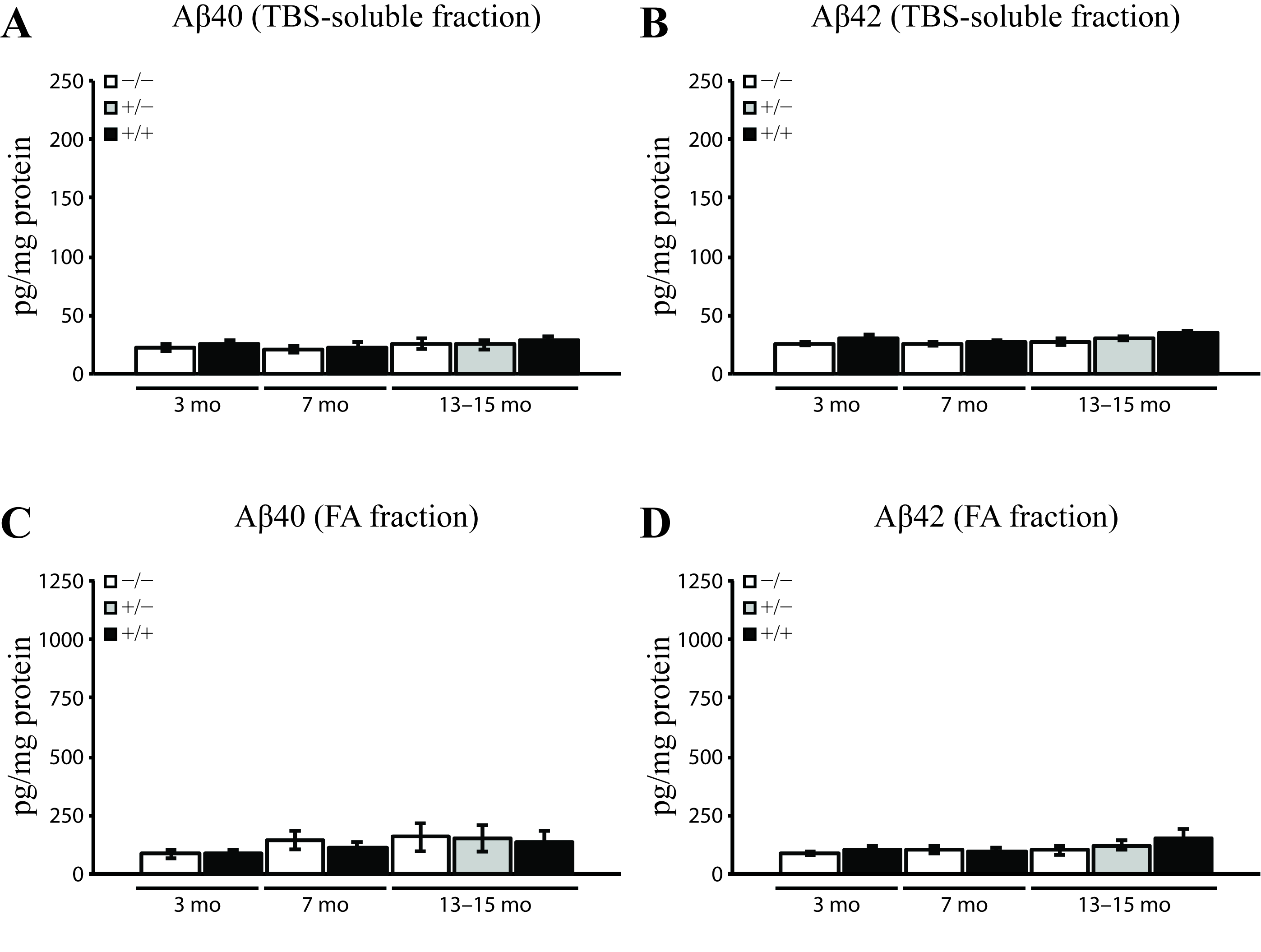

Supplement: Supplementary file 5 — Additional file 5: Figure S4: Quantification of Aβ40 and Aβ42 levels by ELISA in cerebellum. Aβ40 and Aβ42 levels in cerebellum homogenates from non-transgenic (-/-), heterozygous (+/-) and homozygous transgenic (+/+) rats at different ages (3 months, 7 months and 13–15 months) were quantified with specific G2-10/W0-2 and G2-13/W0-2 sandwich ELISAs, respectively. Values were normalized to total protein concentration and expressed as means ± SEM. One-way ANOVA, followed by Dunnett’s post-hoc test. (TIFF 23 MB) [file 40478_2014_135_MOESM5_ESM.tiff]

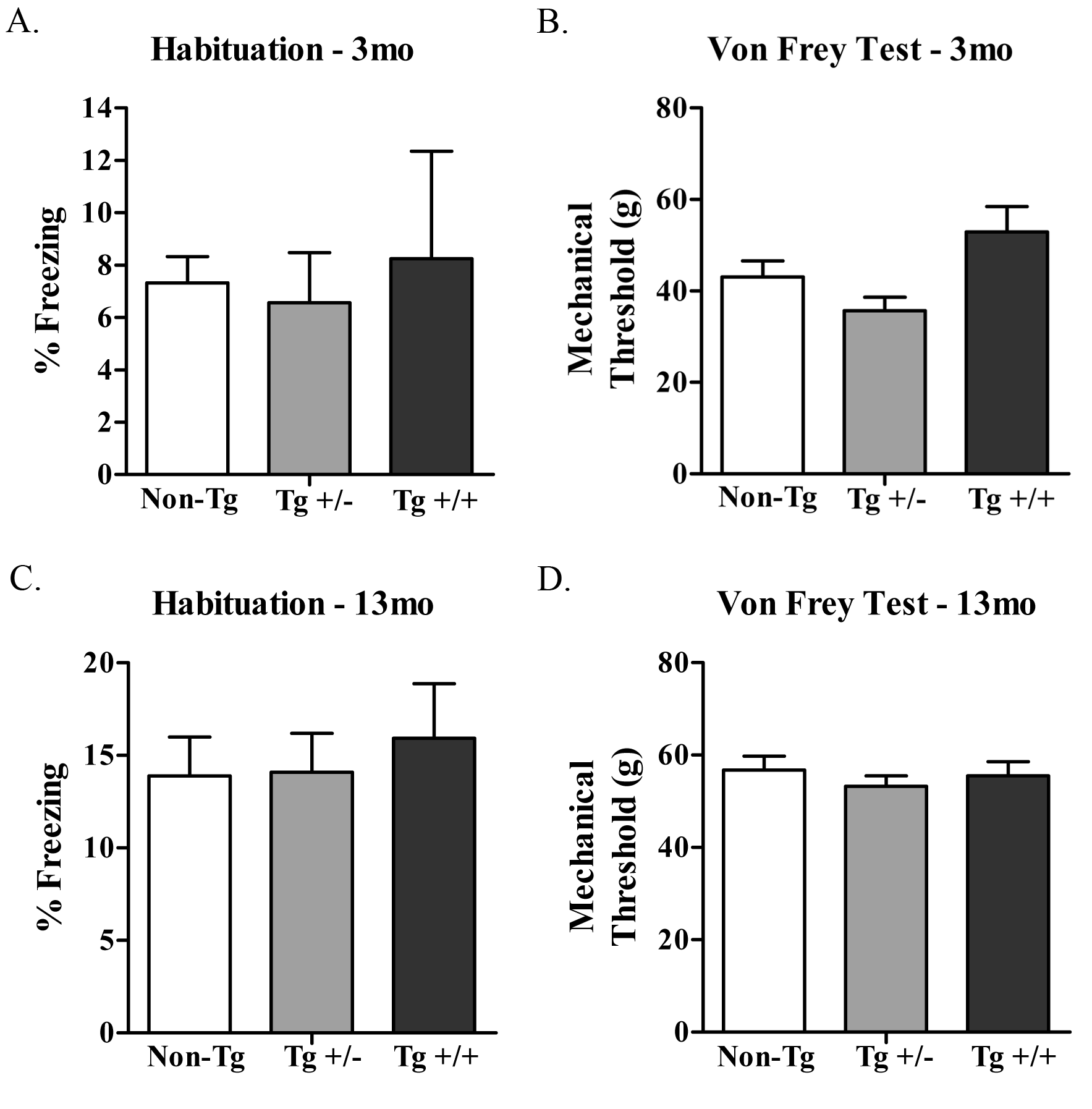

Supplement: Supplementary file 6 — Additional file 6: Figure S5: Analysis of locomotor activity and pain sensitivity in McGill transgenic rats. a, c) Freezing responses recorded during a 5 min exploratory session in the testing environment. The animals were allowed to explore the arena and returned to their home cages. No stimuli were presented. b, d) Analysis of tactile sensitivity in the rat hind paw using Von Frey filaments of increasing force. The graph depicts the average withdrawal thresholds to mechanical stimulation, expressed in grams of pressure. Data is expressed as mean ± SEM. One-Way ANOVA, followed by Bonferroni post-hoc tests. (TIFF 3 MB) [file 40478_2014_135_MOESM6_ESM.tiff]
